# Isolation and antibiotic sensitivity of *Campylobacter* species from fecal samples of broiler chickens in North West Province, South Africa

**DOI:** 10.14202/vetworld.2021.2929-2935

**Published:** 2021-11-21

**Authors:** Kealeboga Mileng, Tsepo A. Ramatla, Rendani V. Ndou, Oriel M. M. Thekisoe, Michelo Syakalima

**Affiliations:** 1 Department of Animal Health, School of Agriculture, North-West University, Private Bag X2046, Mmabatho, 2735, South Africa; 2 Unit for Environmental Sciences and Management, North-West University, Private Bag X6001, Potchefstroom 2531, South Africa

**Keywords:** antibiotic resistance, *Campylobacter jejuni*, chickens, South Africa

## Abstract

**Background and Aim:**

Infections with *Campylobacter* species have gained recognition as the most frequent cause of foodborne gastroenteritis globally. Their significance in South Africa is still an area of study interest. This study was, therefore, carried out to determine the occurrence of *Campylobacter* species in chickens from North West Province of South Africa as well as their antibiotic sensitivity status.

**Materials and Methods:**

A total of 2400 chicken fecal samples were collected and pooled to a total of 480 samples from five registered active poultry abattoirs in the Ngaka Modiri Molema District of North West Province, South Africa. Polymerase chain reaction (PCR) was used for the detection of *Campylobacter* spp. targeting the *16S* *rRNA* gene while antibiotic sensitivity was determined using disk diffusion inhibition test.

**Results:**

After isolation, a total of 26 samples were confirmed to be harboring *Campylobacter jejuni* by PCR and sequencing. *C. jejuni* was found to be the only isolate detected in all the fecal samples tested. The study further demonstrated that *C. jejuni* infections were highest in the summer season (3%) followed by autumn and winter at 1%, while there were none detected in the spring. The isolated *C. jejuni*-positive samples on disk diffusion inhibition test displayed resistance to nalidixic acid, tetracycline, erythromycin, and ciprofloxacin at 98%, 80%, 83%, and 21%, respectively.

**Conclusion:**

*C. jejuni* isolated in this study is known to cause disease in humans, and thus its occurrence requires application of “One Health” strategy to reduce the spread of this zoonotic pathogen in South Africa.

## Introduction

Foodborne diseases result from the consumption of food that is contaminated with pathogens which include bacteria, viruses, and parasites [1]. Outbreaks and sporadic cases of foodborne diseases are common worldwide [2]. One such infection is by bacteria of the genus *Campylobacter* which has gained recognition as the most frequent cause of global foodborne bacterial gastroenteritis overtaking those of *Escherichia coli* and *Salmonella* species in humans [3,4]. *Campylobacter* spp. which are commonly documented as the main contributors to foodborne diseases in humans are *Campylobacter jejuni* and *Campylobacter coli* [5,6].

Poultry is regarded as reservoir of *Campylobacter* spp., and it has been reported that up to a 100% of chickens at slaughter age may be infected [7]. However, chickens are considered as the main reservoir of *Campylobacter* spp. that leads to human campylobacteriosis, *Campylobacter* spp. can colonize chickens without causing any clinical signs in them [8]. In chickens, *Campylobacter* spp. are mostly found in the intestines, mainly in cecal and cloacal crypts [9,10]. However, the organism may be recovered from various organs, including the liver, small intestines, and gizzard [10]. As a result, it has become the major source of poultry meat and poultry product contamination that can lead to food safety concerns. Prevalence rates of *Campylobacter* spp. infection in slaughter age broiler flocks can be as high as 100% on some farm settings [11], thus making fecal contamination of poultry meat during slaughter a high-risk point in the transmission cycle of the disease to consumers.

Despite interest in studying this pathogen, a number of limitations still exist and the most important of which arise from the difficulty in culturing/and or isolating the organism. The types of *Campylobacter* spp. that can be isolated in the laboratory by culture procedures are influenced by the type of media used, culture method, and also the time and conditions from sample collection to culture [12]. It is difficult to detect and identify *Campylobacter* spp. due to its unique culture requirements as well as long incubation period [13,14]. However, different researchers have been using different methods to detect *Campylobacter* spp. including quantitative polymerase chain reaction (qPCR) [15], immunohistochemistry, fluorescence *in situ* hybridization [16], colorimetric aptasensor [17], and immunochromatographic assay [18].

Apart from knowing the diversity of *Campylobacter* spp. at slaughterhouses that may pose a risk to humans, there is also a growing concern about how this *Campylobacter* spp. respond to treatment with antimicrobial substances. The improper use of antimicrobial agents in food animals has resulted in the exposure and circulation of antimicrobial resistance bacteria, including antimicrobial-resistant *Campylobacter* [19,20]. Even though the resistance of *Campylobactor spp*. To antimicrobial agents has been reported worldwide, the situation seems to be graver and accelerating more rapidly in developing countries, where there is uncontrolled and widespread use of antibiotics [21]. It is important to document information on the occurrence as well as the development of antibiotic resistance by local isolates of *Campylobacter* spp. of each country.

Hence this study sought to characterize *Campylobacter* spp. found in feces of slaughter age broiler chickens at Ngaka Modiri Molema District Municipality of North West Province, South Africa, and further evaluated their antibiotic resistance status.

## Materials and Methods

### Ethical approval

The animal and human experimentation and animal care procedures ethical committee of NWU approved the study (Ethics number: NWU-00511-18-A5).

### Study period and location

**Figure-1:**
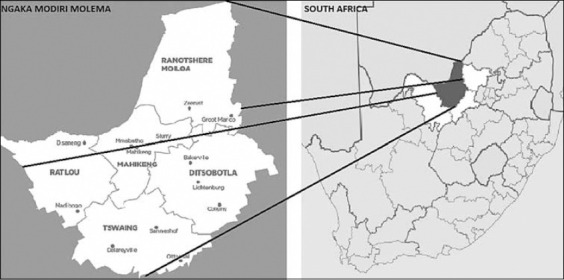
Map of South Africa showing sampling area in the Ngaka Modiri Molema District of North West Province, South Africa [Source: https://showme.co.za/facts-about-south-africa/the-maps-of-south-africa].

The study was conducted from September 2017 to July 2018. This study was conducted around Mafikeng city in the Ngaka Modiri Molema district of the North West Province, South Africa (Figure-1). The province is the second-largest chicken producer in South Africa at 21.3% after Western Cape with 21.9% according to South African Poultry Association [http://www.sapoultry.co.za/pdf-news/sapa-survey-report-role-and-function-2014.pdf].

### Sample collection

A total of 2400 chicken fecal samples were collected randomly post-evisceration from the intestines of randomly selected broiler chickens at slaughterhouses. Feces were picked from the caeca/rectum and were placed into sterile fecal containers. Each container consisted of pooled feces from five different broiler chickens of the same farm, resulting in an overall total of 480 pooled samples (Table-1). All samples were placed on ice and immediately transported to the laboratory of the Centre for Animal Health Studies of North West University for analysis.

**Table-1:** The number of samples collected and the total pooled samples.

Season	Collected samples	Pooled samples
Summer	600	(600/5) 120
Autumn	600	(600/5) 120
Winter	600	(600/5) 120
Spring	600	(600/5) 120
Total	2400	(20,400/5) 480

### Enrichment and culture of Campylobacter species

Fecal samples were cultured within an hour of collection onto a *Campylobacter* selective media to isolate and identify the bacteria for antimicrobial sensitivity testing [22]. Approximately 0.2 g of feces was added to 1 mL blood free *Campylobacter* selective broth and incubated at 5°C in a microaerophilic atmosphere for 48 h. The broth was then vortexed at 14,000 rpm for 3 min and plated onto *Campylobacter* blood-free selective agar base (Modified CCDA-Preston), which is supplemented with CCDA selective supplement SR0155E (Oxoid England). Inoculated plates were incubated at 41.5°C in the microaerophilic atmosphere for 48 h. Suspect colonies were subcultured on *Campylobacter* blood-free selective agar base (Modified CCDA-Preston) to get pure cultures.

### Molecular analysis

The total genomic DNA of cultivated isolates was purified following Zymo Research Fungal/Bacterial DNA kit instructions (Zymo Research Corp., CA, USA). A final, 100 μL of DNA elution buffer was added to elute the DNA which was quantified using a Nanodrop (Bio-Rad Incorporated, CA, USA).

Conventional PCR was used to detect *Campylobacter* spp. in the chicken feces using universal 16S *rRNA* *Campylobacter* spp. primers, namely, the forward primer C412F: GGA TGA CAC ACT TTT CGG AGC and the reverse C1228R: CAT TGT AGC ACG TGT GTC. DNA extracted from chicken fecal samples was subjected to PCR according to conditions described by Bullman *et al*. [23] using Engine DYAD Peltier thermal cycler (Bio-Rad, USA). A total reaction volume of 25 μL containing 3 μL DNA template, 12.5 μL PCR Master Mix, 8.5 μL nucleus-free water, and 2 μL of primer mix (10 μM each) was used. Amplified PCR products were resolved on a 1.5% agarose gel stained with ethidium bromide and visualized with Syngene InGenius Bioimager (UK).

The PCR products were sent for sequencing at Inqaba Biotechnical Industries (Pty) Ltd., Pretoria, South Africa. The homology of partial sequences obtained was compared with the sequences from the NCBI GenBank using nucleotide Basic Local Alignment Search Tool (BLASTn) and nucleotide sequences with similarity above 95% were considered as accurate.

### Antibiotic susceptibility testing of *Campylobacter* isolates

Phenotyping antibacterial susceptibility screening to ciprofloxacin (5 μg), nalidixic acid (30 μg), erythromycin (15 μg), and tetracycline (30 μg) was conducted as recommended by the World Health Organization (WHO) Advisory Group on Integrated Surveillance of Antimicrobial Resistance guidelines on foodborne bacteria. The test was performed using the Kirby–Bauer disk diffusion method and the results were interpreted using the Clinical and Laboratory Standards Institute (CLSI) guidelines [24]. Antibacterial susceptibility test was performed on Mueller-Hinton agar (Neogen Corporation, Lansing, MI, United States) that was supplemented with 10% sheep blood, according to CLSI guidelines. The plates were then sealed in an anaerobic jar 2.5L (Oxoid, UK) each containing gas generating sachet without catalyst in a microaerophilic incubator at 41°C for 24 h. The suspect colonies were subjected to Gram staining and examination under a light microscope for the identification of any potential *Campylobacter* spp. Antibiotic susceptibility was calculated by the zones of inhibition observed around each antibiotic disc in millimeters. Standard reference strains of *Staphylococcus aureus* (ATCC^®^ 29213, Thermo Fischer, USA) and *C. jejuni* ATCC (33560) were used as quality controls [22,25].

## Results

A total of 480 samples were cultured on *Campylobacter* blood-free selective agar base (Modified CCDA-Preston). Colonies of typical Gram-negative curved rods were observed showing an “S” formation. Out of 480 samples, only 336 were suspected to be *Campylobacter* spp. The DNA extraction carried out from the identified colonies and PCR resulted in 70% (336/480) showing 816 bp *Campylobacter* spp.-like bands. However, after sequencing, only 26 PCR products generated nucleotide sequences which perfectly matched with *C. jejuni* using BLASTn and the sequences were submitted to the National Center for Biotechnology Information GenBank database (www.ncbi.nlm.nih.gov/BLAST) and assigned accession numbers, as shown in Table-2. The percentage of *Campylobacter* recovery was highest in summer and lowest in spring. The percentage positive, *Campylobacter* isolates, and seasons of fecal samples used in this study are summarized in Table-3.

**Table-2:** Results of 26 isolates for 16S *rRNA* sequencing (PCR) and accession number.

Samples ID	Sequence ID	Reference from NCBI database	Accession number in GenBank	Assigned accession number	Percentage similarity (%)
NWU1	Seq1	*Campylobacter jejuni*	LC382117	MZ209102	100
NWU2	Seq2	*Campylobacter jejuni*	CP028909	MZ209103	99
NWU3	Seq3	*Campylobacter jejuni*	KY559041	MZ209104	99
NWU4	Seq4	*Campylobacter jejuni*	CP022079	MZ209105	99
NWU5	Seq5	*Campylobacter jejuni*	KY559043	MZ209106	99
NWU6	Seq6	*Campylobacter jejuni*	CP028912	MZ209107	100
NWU7	Seq7	*Campylobacter jejuni*	CP028185	MZ209108	99
NWU8	Seq8	*Campylobacter jejuni*	CP022079	MZ209109	99
NWU9	Seq9	*Campylobacter jejuni*	LC382117	MZ209110	99
NWU10	Seq10	*Campylobacter jejuni*	MF872610	MZ209111	99
NWU11	Seq11	*Campylobacter jejuni*	CP028912	MZ209112	99
NWU12	Seq12	*Campylobacter jejuni*	CP022079	MZ209113	99
NWU13	Seq13	*Campylobacter jejuni*	KY559041	MZ209114	99
NWU14	Seq14	*Campylobacter jejuni*	CP022079	MZ209115	99
NWU15	Seq15	*Campylobacter jejuni*	CP028912	MZ209116	99
NWU16	Seq16	*Campylobacter jejuni*	KY559041	MZ209117	99
NWU17	Seq17	*Campylobacter jejuni*	CP023866	MZ209118	98
NWU18	Seq18	*Campylobacter jejuni*	KY559041	MZ209119	99
NWU19	Seq19	*Campylobacter jejuni*	CP028185	MZ209120	99
NWU20	Seq20	*Campylobacter jejuni*	MF872610	MZ209121	99
NWU21	Seq21	*Campylobacter jejuni*	CP028912	MZ209122	98
NWU22	Seq22	*Campylobacter jejuni*	CP028909	MZ209123	99
NWU23	Seq23	*Campylobacter jejuni*	CP022079	MZ209124	99
NWU24	Seq24	*Campylobacter jejuni*	KY559041	MZ209124	99
NWU25	Seq25	*Campylobacter jejuni*	CP028912	MZ209126	100
NWU26	Seq26	*Campylobacter jejuni*	MF872610	MZ209127	100

PCR=Polymerase chain reaction

**Table-3:** Seasonal patterns of isolated *Campylobacter* spp. in chicken feces.

Season	Collected samples	Total pooled samples	Positive samples	*Campylobacter* spp. (%)	*Campylobacter jejuni* (%)
Summer	600	(600/5) 120	144/480	14/480 (3)	14/480 (3)
Autumn	600	(600/5) 120	96/480	6/480 (1)	6/480 (1)
Winter	600	(600/5) 120	96/480	6/480 (1)	6/480 (1)
Spring	600	(600/5) 120	0	0	0
Total	2400	(2400/5) 480	336/480	26/480 (5)	26/480 (5)

### Antibiotic susceptibility

**Figure-2:**
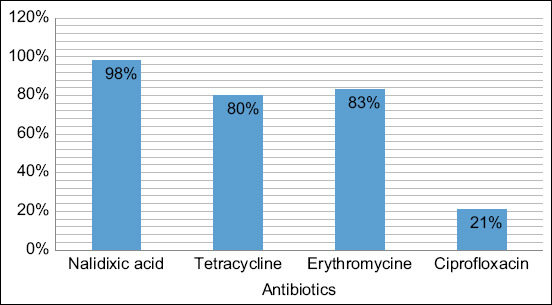
Antimicrobial resistance of *Campylobacter* isolated from fecal samples of broiler chickens.

The antibiotic susceptibility profiles of *C. jejuni* showed a high percentage resistance to nalidixic acid at 98%, tetracycline at 80%, erythromycin at 83%, and the lowest percentage resistance was ciprofloxacin at 21%. The antibiotics and concentrations used in this study, as well as the interpretation of results obtained, are listed in Figure-2. Multidrug resistance to more than 2 classes of antibiotics was found in 4 (15%) isolates.

## Discussion

This study investigated *Campylobacter* spp. diversity in slaughter age chickens. *C. jejuni* (7.7%) was the only *Campylobacter* spp. isolated. This is a very significant finding since this is an important zoonotic infection under the “One health” paradigm, as it causes disease to humans which is acquired through chicken product consumption. The study, therefore, highlights that *C. jejuni* is the dominant species of the foodborne bacteria of the genus *Campylobacter* occurring in Mafikeng. This observation may, however, also mean that the method of isolation and/or sample processing we used was not adequate enough to pick up the other *Campylobacter* species in the samples collected. This can be seen from the number of *Campylobacter*-like bands that were seen after PCR on the gel, but which were not identified as such after sequencing. It is known that the time it takes to collect the sample and final transportation to the laboratory greatly affects the yield of *Campylobacter* diverse species.

Poultry meat and products have been considered a major source of *C. jejuni* for humans [26] and this study has also confirmed this likely source of transmission. The contamination of the meat and products with fecal matter containing *C*. *jejuni*, a known human pathogen, occurs during the slaughter process, especially at the time of degutting. The consumption of undercooked poultry meat and its products can thus be a major cause of human infections [27]. Furthermore, cross-contamination in the kitchen from contaminated meat to other food items, especially those that will not be cooked, is also considered a major pathway of transmission [27,28]. Although this study did not explore that route of transmission in the kitchen, the presence of contamination of the carcasses at slaughter makes this cross-contamination very possible.

A study by Richardson *et al*. [29] conducted in Soweto, South Africa, reported the relative ease of *C. jejuni* acquisition by families. This was attributed to the fact that poultry meat and meat products are highly consumed and more available because they are cheap and are also farmed in the backyards in different communities [29]. In Kwazulu-Natal Province, 43% of *C. jejuni* was confirmed [30]. In the study conducted by Richardson *et al*. [29], *C. jejuni* was recovered in 86% of fowl feces, pet dog feces, and bovine intestine (another popular food source). A previous study by Samie *et al*. [31] reported prevalence of 10.2% and 6.5% for *C. jejuni* and *C. coli*, respectively, from stool samples of hospital patients and asymptomatic pupils in Venda, Limpopo Province, South Africa. Another study conducted by Lastovica [32] in Cape Town, Western Cape Province, South Africa, detected 40%, 7.7%, and 24.6% of *C. jejuni*, *C. coli*, and *Campylobacter concisus* infections from diarrheagenic stools of children using culture methods.

In the current study, 3% (n=14) of total *C. jejuni* was isolated and confirmed during summer, 1% (n=6) in autumn, and 1% (n=6) in winter and there was no detection of *C. jejuni* during spring. Reports from other studies indicate that infections of *Campylobacter* are mostly sporadic and occur during warmer months of the summer and autumn. Various countries have indicated seasonal patterns to the pathogen, such as New Zealand and Australia [33]. In South Africa, there are limited data on the seasonality of *Campylobacter* spp. infections. This is probably because South Africa, as a developing country, lacks resources to investigate the pathogen on a continuous basis. This statement is supported by Coker *et al*. [34], who stated that public health infrastructure contributes to the lack of data in developing countries. Plats-Mills and Kosek [35] speculated that a lack of data in this regard could be due to the fact that the temperature variations in some developing countries are not as extreme as in developed countries. Nonetheless, the fact that *Campylobacter* is difficult to isolate is the most likely reason for the lack of data. However, the findings in the present study seem to indicate that the summer and warmer months have a higher risk because *C. jejuni* was isolated in more chicken feces during that period. The study area (North West Province) is a semi-desert area with extreme temperatures during summer. Another reason could be that there is less ventilation in summer, so chickens tend to be more stressed and shed more bacteria during this season.

In this study, *C. jejuni* was found to be resistant to nalidixic acid at 98%, tetracycline at 80%, erythromycin at 83%, and ciprofloxacin at 21%. Studies in other countries also show that *Campylobacter* spp. have developed resistance to antibiotics, specifically to macrolide antibiotics and fluoroquinolones, globally [36]. In another study in South Africa, ciprofloxacin resistance in clinical *C. jejuni* isolates from commercial chicken increased from 1.4% to 79% in 14 years between 1998 and 2011 [37]. Furthermore, *C. jejuni* isolated from our study was 80% resistant to tetracycline. According to Basardien [37], tetracycline resistance in *C. jejuni* isolated in commercial chicken increased from 14.2% to 86% between the years 1998 and 2011.

In the present study, *C. jejuni* isolates were 93% resistant to erythromycin. Macrolide antibiotics, including erythromycin, are considered the first drug of choice for human campylobacteriosis cases [38-40]. The resistance of clinical *C. jejuni* isolated in commercial chicken to erythromycin has increased from 3.4% and 97% between the years 1998 and 2012 [37]. However, the recent study conducted by Wieczorek *et al*. [41] reported 0% resistance to erythromycin for *Campylobacter* isolates in Poland. Erythromycin is generally used as the first drug of choice for treating *Campylobacter* gastroenteritis [4,20].

Investigation of the 26 *C. jejuni* isolates for antibiotic resistance revealed that 80% of the isolates were resistant to tetracycline, which is an antibiotic used against both Gram-negative and Gram-positive bacteria, including some other atypical and non-infectious microorganisms through inhibition of protein synthesis in these harmful agents [42,43]. Tetracycline is one of the most commonly used drugs in the livestock industry and it is, therefore, not surprising that one would find the levels of resistance revealed by our study.

The lowest resistance was noticed for ciprofloxacin at 21%. Similar results were obtained from different studies conducted by Geissler *et al*. [44] and Cody *et al*. [45], which reported 9% and 25% ciprofloxacin resistance by *C. jejuni* in the United States and Ireland, respectively.

In this study, multidrug resistance patterns were also observed among the isolates. Our findings do not stand alone since similar findings were observed in Poland, where 321 (60.9%) *Campylobactor spp*. Isolates from poultry were showing multidrug resistance [41], confirming that there is a disturbing increase in the emergence of multidrug-resistant *Campylobacte*r [36,46,47]. Once again this observation of development of antibiotic resistance is of major concern, which is listed by the WHO as an item that requires “One Health” approach (https://www.who.int/news-room/q-a-detail/one-health).

This study is one of the many that has highlighted increased antibiotic resistance build-up in South Africa because of high levels of antibiotic use as therapeutic agents and growth promoters in the South African poultry industry. This has an impact on human health as antimicrobials are used to treat infections that cause morbidities and mortalities. Increased antibiotic resistance decreases the effectiveness of these drugs and results in human health being compromised [48].

## Conclusion

This study successfully isolated *Campylobacter* spp. using direct DNA extraction from chicken feces and broth enrichment approach. The common species occurring in the study area is *C. jejuni* and its highest prevalence was during summer. This suggests that a high risk of contracting campylobacteriosis due to *C. jejuni* could possibly be during this season and chicken meat may be an important source. This study also revealed that the species of *C. jejuni* isolated from chicken feces had an increased prevalence of antibiotic resistance to ciprofloxacin, nalidixic acid, tetracycline, and erythromycin, which are used in the therapeutic treatment of humans that have acquired serious cases of campylobacteriosis. Detection of zoonotic *C. jejuni* and its development of antibiotic resistance highlights the need for “One Health” approach to combat the spread of these bacteria from the environment, animals, and humans in Mafikeng and South Africa as a whole.

## Authors’ Contributions

MS and RVN: Conceived and designed the study and provided reagents and materials. KM: Performed the experiments and wrote the first draft. TAR: Supervised the molecular analyses. OMMT: Analyzed the data, interpreted the results, and reviewed and edited the manuscript. All authors read and approved the final manuscript.
